# ECMO cannula-associated infections: interest of cannula swab and subcutaneous needle aspirate samples for prediction of cannula tip culture

**DOI:** 10.1186/s40635-020-00327-x

**Published:** 2020-07-23

**Authors:** Hadrien Winiszewski, Charles Boyadjian, Guillaume Besch, Andrea Perrotti, Gaël Piton

**Affiliations:** 1 Medical Intensive Care Unit, Besançon University Hospital, Besançon, France; 2 EA3920, University of Franche Comté, Besançon, France; 3 Surgical Intensive Care Unit, Besançon University Hospital, Besançon, France; 4 Cardiac Surgery Unit, Besançon University Hospital, Besançon, France

**Keywords:** ECMO, Cannula infection, Diagnosis

To the Editor,

In a recent review published in Intensive Care Medicine, Abrams et al. listed the research agenda of extracorporeal membrane oxygenation (ECMO)-associated infections. One of the questions was “how should cannula-associated infection be defined?” [1].

Literature on ECMO cannula-associated infections is very scarce [2, 3], and no clear definition is available. Then, diagnostic workup of cannula-related infections is currently derived from central catheter-related infection guidelines. For catheter-related infection, a positive catheter tip culture is a diagnostic criterion [4]. However, this attitude cannot be fully transposed to ECMO cannula-related infections, as cannula cannot be easily removed and changed for tip culture. We therefore aimed at evaluating the performance of cannula swab and subcutaneous needle aspirate for predicting the results of the cannula’s tip culture.

In this prospective observational pilot study, patients treated by ECMO at the Besancon University Hospital (France) were enrolled. When patients had sepsis or local signs of cannula-associated infection, cannula swab and subcutaneous needle aspirate (Fig. 1d) of both arterial and venous cannulas were systematically obtained for culture. For subcutaneous needle aspirate, after cleaning with an antiseptic solution, a catheter for peripheral venous access was inserted on 1 to 3 cm along the ECMO cannula. If no exudate was aspirated, 1 mL of saline was injected and re-aspirated. The aspirate was sent for bacterial culture. Quantitative culture was not performed, and all positive cultures were considered. At the time of ECMO weaning, the cannula tips were also collected for culture. Culture of cannula swab and subcutaneous needle aspirate were considered as “positive AND fully correspondent” with the cannula tip if it detected all the pathogens of the cannula tip. Culture of cannula swab and subcutaneous needle aspirate were considered as “positive AND not fully correspondent” if the culture was positive but some pathogens were lacking or were different.

**Fig 1:**
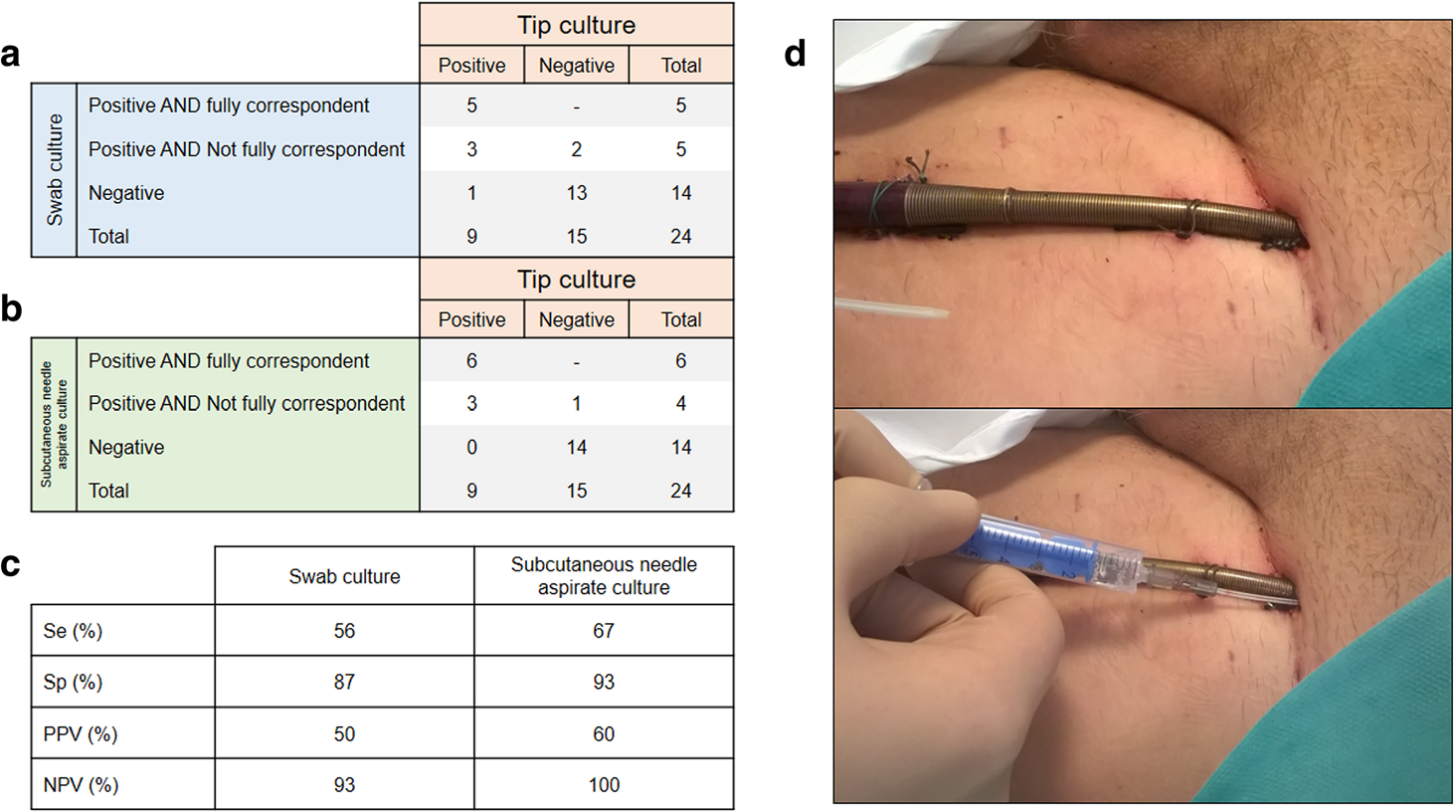
**a** Correspondence between cannula swab culture and cannula tip culture. **b** Correspondence between cannula subcutaneous needle aspirate culture and cannula tip culture. **c** Performance of swab and subcutaneous needle aspirate culture for prediction of cannula tip culture. **d** Example of cannula subcutaneous needle aspirate. Se, sensibility; Sp, specificity; PPV, positive predictive value; NPV, negative predictive value

We included 24 patients, aging 64 years old [IQR 53–70], 71% males, with 21 VA and 3 VV ECMO. Cannulation technique was mostly surgical (20/24), and 19 patients had both cannulas in the same groin. Nine patients (38%) had a positive tip culture, including 5 with associated bacteremia to the same pathogen. Among them, culture was positive for both of the cannula’s tips in 7 patients and positive for only one of the cannula’s tip in 2 patients. Enterobacteriaceae and enterococci were the most common pathogens detected on cannula tips. Median duration between ECMO start and local sampling was 9 [4–11] days. Median duration between local sampling and tip culture was 4 [IQR 2–6] days. Sensibility and positive predictive value for cannula tip positive culture were 56 and 50% for swab and 67 and 60% for subcutaneous needle aspirate (Fig. 1a, c). Specificity and negative predictive value for cannula tip positive culture were 87 and 93% for swab and 93 and 100% for subcutaneous needle aspirate (Fig. 1b, c).

Our data suggest that swab or subcutaneous needle aspirate culture might be useful to predict a negative cannula tip culture. Therefore, the combination of both samples, easily obtained on the bedside, might be of interest to rule out ECMO cannula-related infection.

## Data Availability

The datasets used and/or analyzed during the current study are available from the corresponding author on reasonable request.

## Abbreviations

ECMO: Extracorporeal membrane oxygenation
